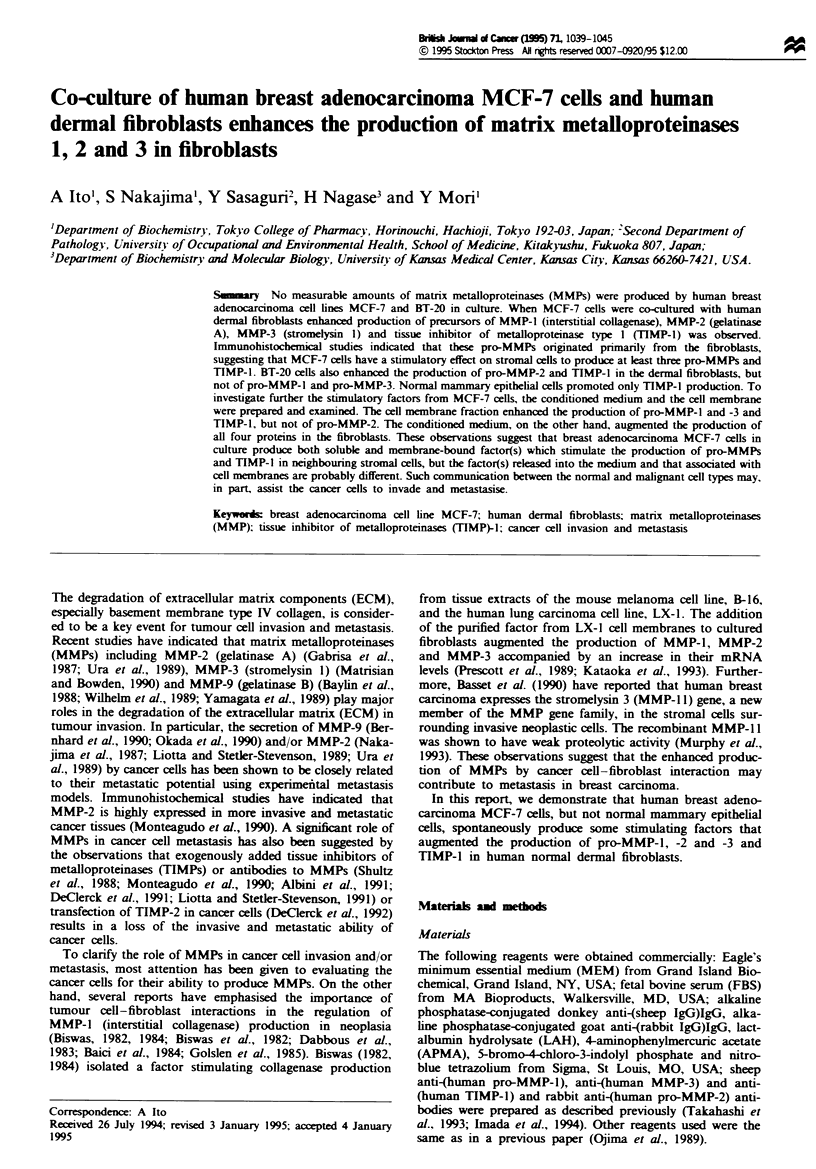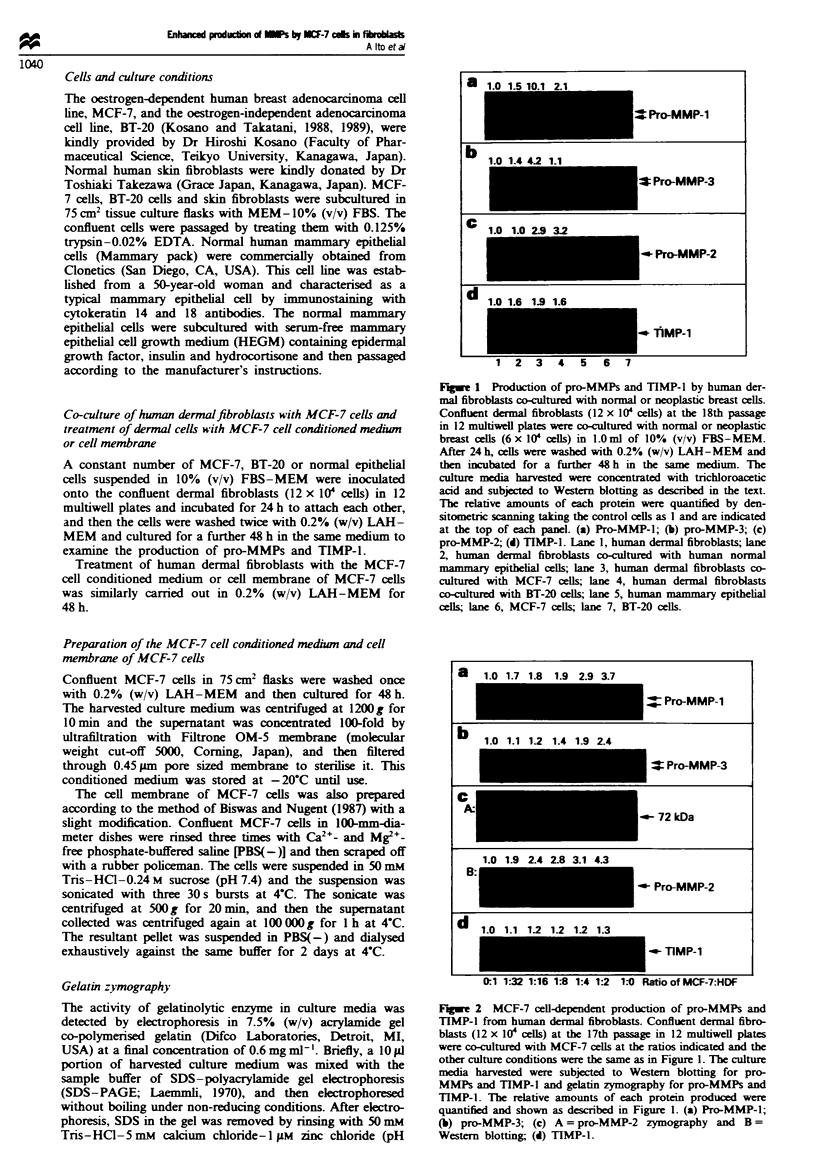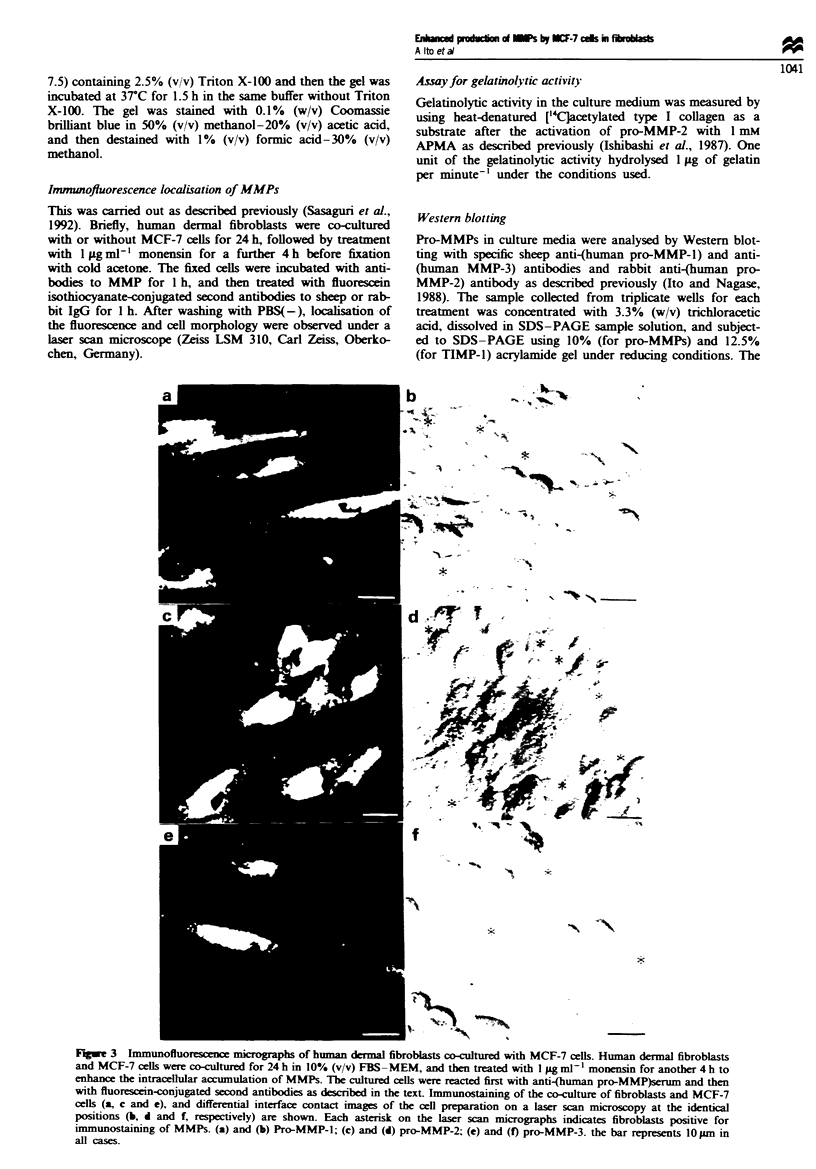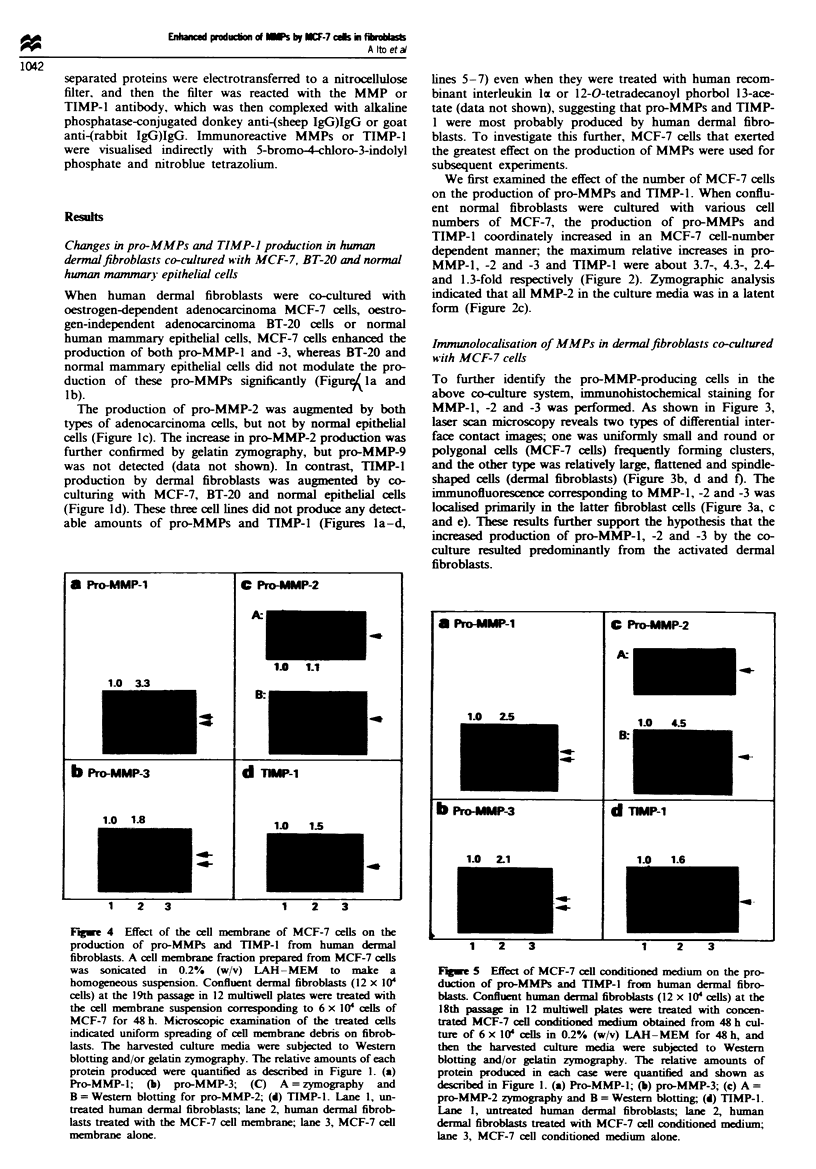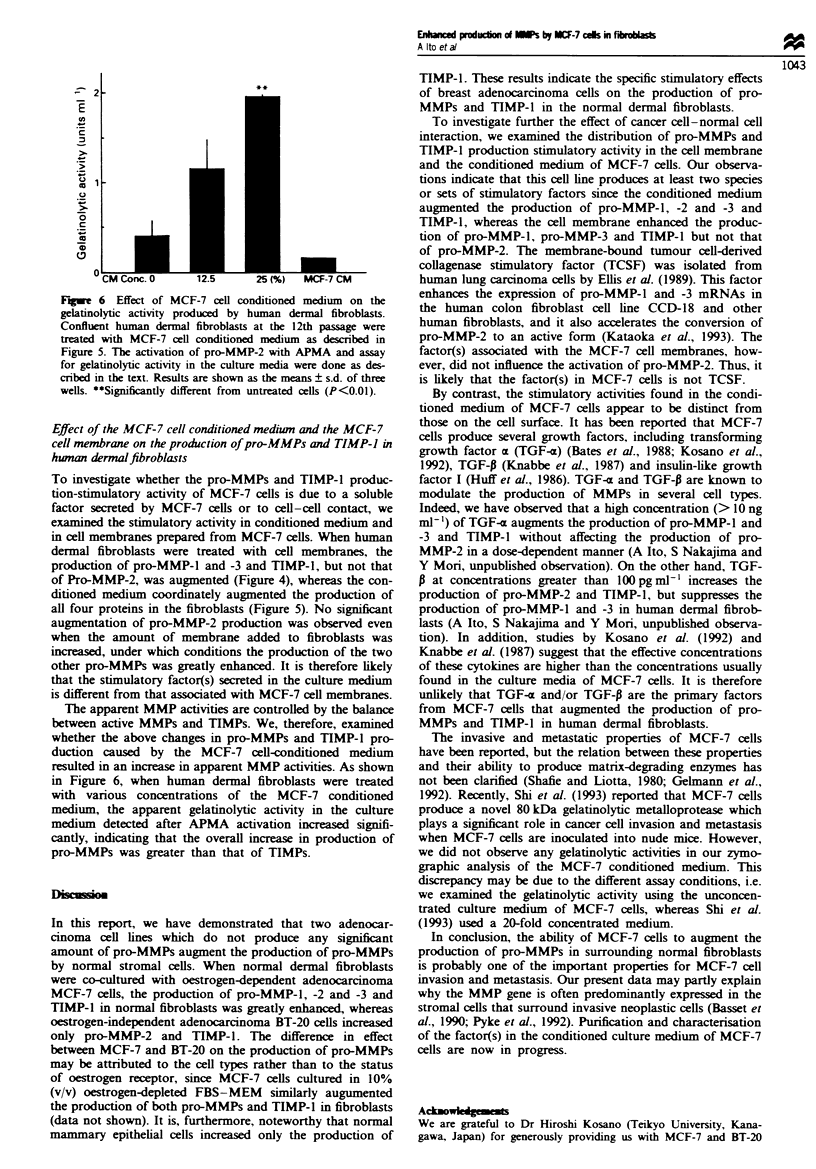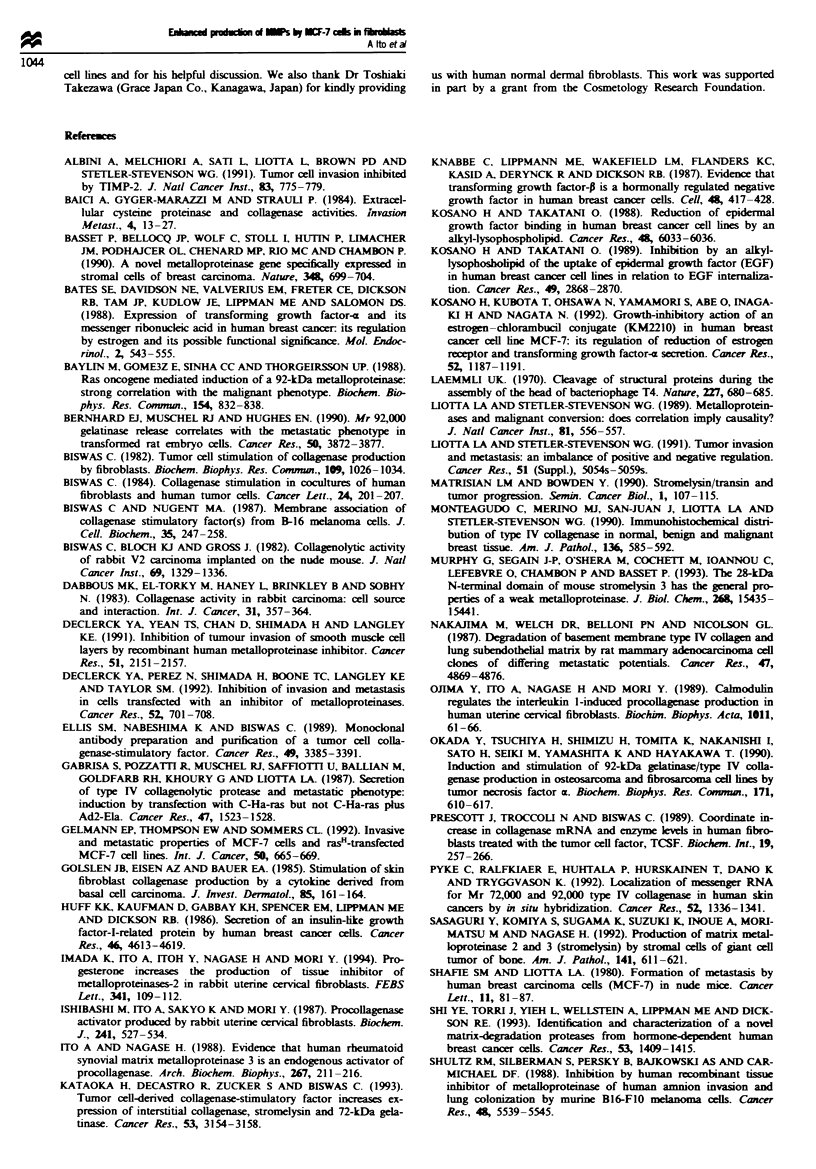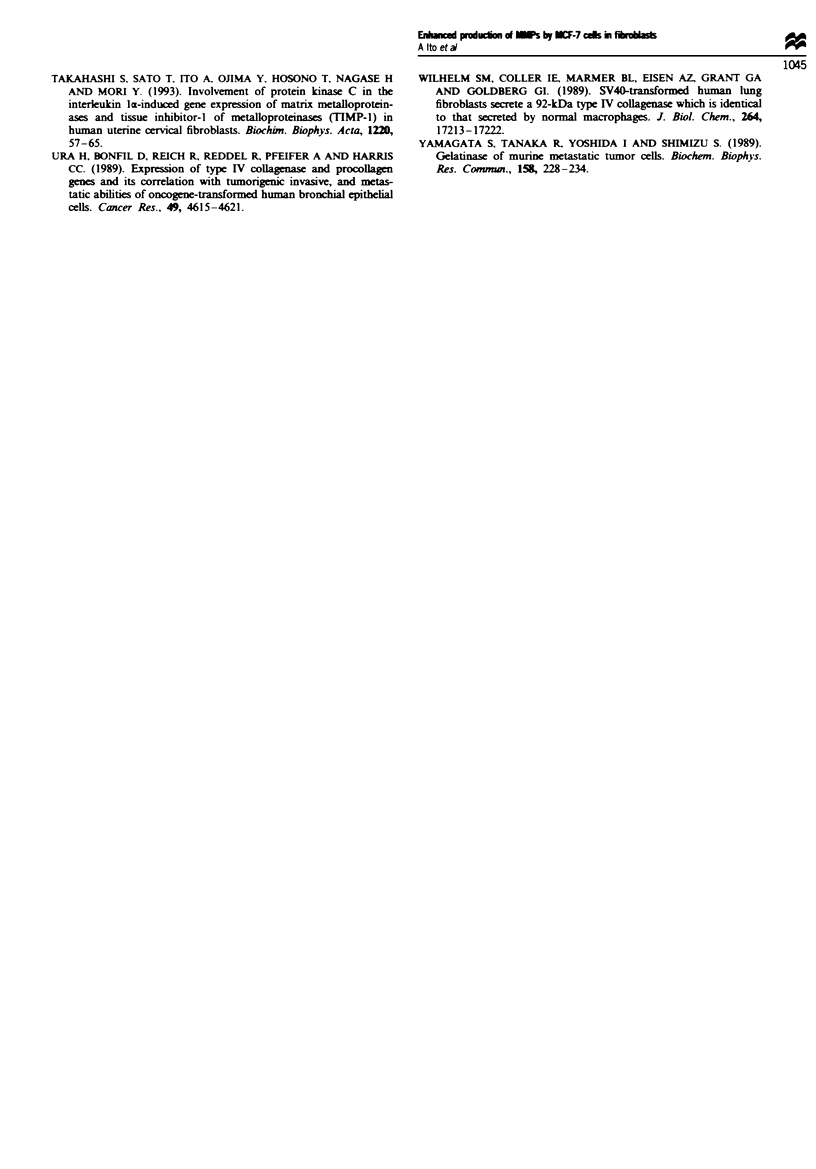# Co-culture of human breast adenocarcinoma MCF-7 cells and human dermal fibroblasts enhances the production of matrix metalloproteinases 1, 2 and 3 in fibroblasts.

**DOI:** 10.1038/bjc.1995.200

**Published:** 1995-05

**Authors:** A. Ito, S. Nakajima, Y. Sasaguri, H. Nagase, Y. Mori

**Affiliations:** Department of Biochemistry, Tokyo College of Pharmacy, Japan.

## Abstract

**Images:**


British Journal of Cancer (1995) 71, 1039–1045

© 1995 Stockton Press All rights reserved 0007–0920/95 $12.00

### Co-culture of human breast adenocarcinoma MCF-7 cells and human dermal fibroblasts enhances the production of matrix metalloproteinases 1, 2 and 3 in fibroblasts

A Ito[1], S Nakajima[1], Y Sasaguri[2], H Nagase[3] and Y Mori[1]

[1]*Department of Biochemistry, Tokyo College of Pharmacy, Horinouchi, Hachioji, Tokyo 192-03, Japan;* [2]*Second Department of Pathology, University of Occupational and Environmental Health, School of Medicine, Kitakyushu, Fukuoka 807, Japan;* [3]*Department of Biochemistry and Molecular Biology, University of Kansas Medical Center, Kansas City, Kansas 66260-7421, USA.*

**Summary** No measurable amounts of matrix metalloproteinases (MMPs) were produced by human breast adenocarcinoma cell lines MCF-7 and BT-20 in culture. When MCF-7 cells were co-cultured with human dermal fibroblasts enhanced production of precursors of MMP-1 (interstitial collagenase), MMP-2 (gelatinase A), MMP-3 (stromelysin 1) and tissue inhibitor of metalloproteinase type 1 (TIMP-1) was observed. Immunohistochemical studies indicated that these pro-MMPs originated primarily from the fibroblasts, suggesting that MCF-7 cells have a stimulatory effect on stromal cells to produce at least three pro-MMPs and TIMP-1. BT-20 cells also enhanced the production of pro-MMP-2 and TIMP-1 in the dermal fibroblasts, but not of pro-MMP-1 and pro-MMP-3. Normal mammary epithelial cells promoted only TIMP-1 production. To investigate further the stimulatory factors from MCF-7 cells, the conditioned medium and the cell membrane were prepared and examined. The cell membrane fraction enhanced the production of pro-MMP-1 and -3 and TIMP-1, but not of pro-MMP-2. The conditioned medium, on the other hand, augmented the production of all four proteins in the fibroblasts. These observations suggest that breast adenocarcinoma MCF-7 cells in culture produce both soluble and membrane-bound factor(s) which stimulate the production of pro-MMPs and TIMP-1 in neighbouring stromal cells, but the factor(s) released into the medium and that associated with cell membranes are probably different. Such communication between the normal and malignant cell types may, in part, assist the cancer cells to invade and metastasise.

**Keywords:** breast adenocarcinoma cell line MCF-7; human dermal fibroblasts; matrix metalloproteinases (MMP); tissue inhibitor of metalloproteinases (TIMP)-1; cancer cell invasion and metastasis

The degradation of extracellular matrix components (ECM), especially basement membrane type IV collagen, is considered to be a key event for tumour cell invasion and metastasis. Recent studies have indicated that matrix metalloproteinases (MMPs) including MMP-2 (gelatinase A) (Gabrisa *et al.*, 1987; Ura *et al.*, 1989), MMP-3 (stromelysin 1) (Matrisian and Bowden, 1990) and MMP-9 (gelatinase B) (Baylin *et al.*, 1988; Wilhelm *et al.*, 1989; Yamagata *et al.*, 1989) play major roles in the degradation of the extracellular matrix (ECM) in tumour invasion. In particular, the secretion of MMP-9 (Bernhard *et al.*, 1990; Okada *et al.*, 1990) and/or MMP-2 (Nakajima *et al.*, 1987; Liotta and Stetler-Stevenson, 1989; Ura *et al.*, 1989) by cancer cells has been shown to be closely related to their metastatic potential using experimental metastasis models. Immunohistochemical studies have indicated that MMP-2 is highly expressed in more invasive and metastatic cancer tissues (Monteagudo *et al.*, 1990). A significant role of MMPs in cancer cell metastasis has also been suggested by the observations that exogenously added tissue inhibitors of metalloproteinases (TIMPs) or antibodies to MMPs (Shultz *et al.*, 1988; Monteagudo *et al.*, 1990; Albini *et al.*, 1991; DeClerck *et al.*, 1991; Liotta and Stetler-Stevenson, 1991) or transfection of TIMP-2 in cancer cells (DeClerck *et al.*, 1992) results in a loss of the invasive and metastatic ability of cancer cells.

To clarify the role of MMPs in cancer cell invasion and/or metastasis, most attention has been given to evaluating the cancer cells for their ability to produce MMPs. On the other hand, several reports have emphasised the importance of tumour cell–fibroblast interactions in the regulation of MMP-1 (interstitial collagenase) production in neoplasia (Biswas, 1982, 1984; Biswas *et al.*, 1982; Dabbous *et al.*, 1983; Baici *et al.*, 1984; Golslen *et al.*, 1985). Biswas (1982, 1984) isolated a factor stimulating collagenase production from tissue extracts of the mouse melanoma cell line, B-16, and the human lung carcinoma cell line, LX-1. The addition of the purified factor from LX-1 cell membranes to cultured fibroblasts augmented the production of MMP-1, MMP-2 and MMP-3 accompanied by an increase in their mRNA levels (Prescott *et al.*, 1989; Kataoka *et al.*, 1993). Furthermore, Basset *et al.* (1990) have reported that human breast carcinoma expresses the stromelysin 3 (MMP-11) gene, a new member of the MMP gene family, in the stromal cells surrounding invasive neoplastic cells. The recombinant MMP-11 was shown to have weak proteolytic activity (Murphy *et al.*, 1993). These observations suggest that the enhanced production of MMPs by cancer cell–fibroblast interaction may contribute to metastasis in breast carcinoma.

In this report, we demonstrate that human breast adenocarcinoma MCF-7 cells, but not normal mammary epithelial cells, spontaneously produce some stimulating factors that augmented the production of pro-MMP-1, -2 and -3 and TIMP-1 in human normal dermal fibroblasts.

### Materials and methods

### *Materials*

The following reagents were obtained commercially: Eagle's minimum essential medium (MEM) from Grand Island Biochemical, Grand Island, NY, USA; fetal bovine serum (FBS) from MA Bioproducts, Walkersville, MD, USA; alkaline phosphatase-conjugated donkey anti-(sheep IgG)IgG, alkaline phosphatase-conjugated goat anti-(rabbit IgG)IgG, lactalbumin hydrolysate (LAH), 4-aminophenylmercuric acetate (APMA), 5-bromo-4-chloro-3-indolyl phosphate and nitroblue tetrazolium from Sigma, St Louis, MO, USA; sheep anti-(human pro-MMP-1), anti-(human MMP-3) and anti-(human TIMP-1) and rabbit anti-(human pro-MMP-2) antibodies were prepared as described previously (Takahashi *et al.*, 1993; Imada *et al.*, 1994). Other reagents used were the same as in a previous paper (Ojima *et al.*, 1989).

Correspondence: A Ito
Received 26 July 1994; revised 3 January 1995; accepted 4 January 1995

*Cells and culture conditions*

The oestrogen-dependent human breast adenocarcinoma cell line, MCF-7, and the oestrogen-independent adenocarcinoma cell line, BT-20 (Kosano and Takatani, 1988, 1989), were kindly provided by Dr Hiroshi Kosano (Faculty of Pharmaceutical Science, Teikyo University, Kanagawa, Japan). Normal human skin fibroblasts were kindly donated by Dr Toshiaki Takezawa (Grace Japan, Kanagawa, Japan). MCF-7 cells, BT-20 cells and skin fibroblasts were subcultured in 75 $cm^2$ tissue culture flasks with MEM–10% (v/v) FBS. The confluent cells were passaged by treating them with 0.125% trypsin–0.02% EDTA. Normal human mammary epithelial cells (Mammary pack) were commercially obtained from Clonetics (San Diego, CA, USA). This cell line was established from a 50-year-old woman and characterised as a typical mammary epithelial cell by immunostaining with cytokeratin 14 and 18 antibodies. The normal mammary epithelial cells were subcultured with serum-free mammary epithelial cell growth medium (HEGM) containing epidermal growth factor, insulin and hydrocortisone and then passaged according to the manufacturer's instructions.

*Co-culture of human dermal fibroblasts with MCF-7 cells and treatment of dermal cells with MCF-7 cell conditioned medium or cell membrane*

A constant number of MCF-7, BT-20 or normal epithelial cells suspended in 10% (v/v) FBS–MEM were inoculated onto the confluent dermal fibroblasts ($12 \times 10^4$ cells) in 12 multiwell plates and incubated for 24 h to attach each other, and then the cells were washed twice with 0.2% (w/v) LAH–MEM and cultured for a further 48 h in the same medium to examine the production of pro-MMPs and TIMP-1.

Treatment of human dermal fibroblasts with the MCF-7 cell conditioned medium or cell membrane of MCF-7 cells was similarly carried out in 0.2% (w/v) LAH–MEM for 48 h.

*Preparation of the MCF-7 cell conditioned medium and cell membrane of MCF-7 cells*

Confluent MCF-7 cells in 75 $cm^2$ flasks were washed once with 0.2% (w/v) LAH–MEM and then cultured for 48 h. The harvested culture medium was centrifuged at 1200 *g* for 10 min and the supernatant was concentrated 100-fold by ultrafiltration with Filtrone OM-5 membrane (molecular weight cut-off 5000, Corning, Japan), and then filtered through 0.45 μm pore sized membrane to sterilise it. This conditioned medium was stored at −20°C until use.

The cell membrane of MCF-7 cells was also prepared according to the method of Biswas and Nugent (1987) with a slight modification. Confluent MCF-7 cells in 100-mm-diameter dishes were rinsed three times with $Ca^{2+}$- and $Mg^{2+}$-free phosphate-buffered saline [PBS(−)] and then scraped off with a rubber policeman. The cells were suspended in 50 mM Tris–HCl–0.24 M sucrose (pH 7.4) and the suspension was sonicated with three 30 s bursts at 4°C. The sonicate was centrifuged at 500 *g* for 20 min, and then the supernatant collected was centrifuged again at 100 000 *g* for 1 h at 4°C. The resultant pellet was suspended in PBS(−) and dialysed exhaustively against the same buffer for 2 days at 4°C.

*Gelatin zymography*

The activity of gelatinolytic enzyme in culture media was detected by electrophoresis in 7.5% (w/v) acrylamide gel co-polymerised gelatin (Difco Laboratories, Detroit, MI, USA) at a final concentration of 0.6 mg $ml^{-1}$. Briefly, a 10 μl portion of harvested culture medium was mixed with the sample buffer of SDS–polyacrylamide gel electrophoresis (SDS–PAGE; Laemmli, 1970), and then electrophoresed without boiling under non-reducing conditions. After electrophoresis, SDS in the gel was removed by rinsing with 50 mM Tris–HCl–5 mM calcium chloride–1 μM zinc chloride (pH

**Figure 1** Production of pro-MMPs and TIMP-1 by human dermal fibroblasts co-cultured with normal or neoplastic breast cells. Confluent dermal fibroblasts ($12 \times 10^4$ cells) at the 18th passage in 12 multiwell plates were co-cultured with normal or neoplastic breast cells ($6 \times 10^4$ cells) in 1.0 ml of 10% (v/v) FBS–MEM. After 24 h, cells were washed with 0.2% (w/v) LAH–MEM and then incubated for a further 48 h in the same medium. The culture media harvested were concentrated with trichloroacetic acid and subjected to Western blotting as described in the text. The relative amounts of each protein were quantified by densitometric scanning taking the control cells as 1 and are indicated at the top of each panel. (**a**) Pro-MMP-1; (**b**) pro-MMP-3; (**c**) pro-MMP-2; (**d**) TIMP-1. Lane 1, human dermal fibroblasts; lane 2, human dermal fibroblasts co-cultured with human normal mammary epithelial cells; lane 3, human dermal fibroblasts co-cultured with MCF-7 cells; lane 4, human dermal fibroblasts co-cultured with BT-20 cells; lane 5, human mammary epithelial cells; lane 6, MCF-7 cells; lane 7, BT-20 cells.

**Figure 2** MCF-7 cell-dependent production of pro-MMPs and TIMP-1 from human dermal fibroblasts. Confluent dermal fibroblasts ($12 \times 10^4$ cells) at the 17th passage in 12 multiwell plates were co-cultured with MCF-7 cells at the ratios indicated and the other culture conditions were the same as in Figure 1. The culture media harvested were subjected to Western blotting for pro-MMPs and TIMP-1 and gelatin zymography for pro-MMPs and TIMP-1. The relative amounts of each protein produced were quantified and shown as described in Figure 1. (**a**) Pro-MMP-1; (**b**) pro-MMP-3; (**c**) A = pro-MMP-2 zymography and B = Western blotting; (**d**) TIMP-1.

7.5) containing 2.5% (v/v) Triton X-100 and then the gel was incubated at 37°C for 1.5 h in the same buffer without Triton X-100. The gel was stained with 0.1% (w/v) Coomassie brilliant blue in 50% (v/v) methanol–20% (v/v) acetic acid, and then destained with 1% (v/v) formic acid–30% (v/v) methanol.

*Immunofluorescence localisation of MMPs*

This was carried out as described previously (Sasaguri *et al.*, 1992). Briefly, human dermal fibroblasts were co-cultured with or without MCF-7 cells for 24 h, followed by treatment with 1 μg ml$^{-1}$ monensin for a further 4 h before fixation with cold acetone. The fixed cells were incubated with antibodies to MMP for 1 h, and then treated with fluorescein isothiocyanate-conjugated second antibodies to sheep or rabbit IgG for 1 h. After washing with PBS(−), localisation of the fluorescence and cell morphology were observed under a laser scan microscope (Zeiss LSM 310, Carl Zeiss, Oberkochen, Germany).

*Assay for gelatinolytic activity*

Gelatinolytic activity in the culture medium was measured by using heat-denatured [$^{14}$C]acetylated type I collagen as a substrate after the activation of pro-MMP-2 with 1 mM APMA as described previously (Ishibashi *et al.*, 1987). One unit of the gelatinolytic activity hydrolysed 1 μg of gelatin per minute$^{-1}$ under the conditions used.

*Western blotting*

Pro-MMPs in culture media were analysed by Western blotting with specific sheep anti-(human pro-MMP-1) and anti-(human MMP-3) antibodies and rabbit anti-(human pro-MMP-2) antibody as described previously (Ito and Nagase, 1988). The sample collected from triplicate wells for each treatment was concentrated with 3.3% (w/v) trichloracetic acid, dissolved in SDS–PAGE sample solution, and subjected to SDS–PAGE using 10% (for pro-MMPs) and 12.5% (for TIMP-1) acrylamide gel under reducing conditions. The

**Figure 3** Immunofluorescence micrographs of human dermal fibroblasts co-cultured with MCF-7 cells. Human dermal fibroblasts and MCF-7 cells were co-cultured for 24 h in 10% (v/v) FBS–MEM, and then treated with 1 μg ml$^{-1}$ monensin for another 4 h to enhance the intracellular accumulation of MMPs. The cultured cells were reacted first with anti-(human pro-MMP)serum and then with fluorescein-conjugated second antibodies as described in the text. Immunostaining of the co-culture of fibroblasts and MCF-7 cells (**a**, **c** and **e**), and differential interface contact images of the cell preparation on a laser scan microscopy at the identical positions (**b**, **d** and **f**, respectively) are shown. Each asterisk on the laser scan micrographs indicates fibroblasts positive for immunostaining of MMPs. (**a**) and (**b**) Pro-MMP-1; (**c**) and (**d**) pro-MMP-2; (**e**) and (**f**) pro-MMP-3. the bar represents 10 μm in all cases.

separated proteins were electrotransferred to a nitrocellulose filter, and then the filter was reacted with the MMP or TIMP-1 antibody, which was then complexed with alkaline phosphatase-conjugated donkey anti-(sheep IgG)IgG or goat anti-(rabbit IgG)IgG. Immunoreactive MMPs or TIMP-1 were visualised indirectly with 5-bromo-4-chloro-3-indolyl phosphate and nitroblue tetrazolium.

### Results

### *Changes in pro-MMPs and TIMP-1 production in human dermal fibroblasts co-cultured with MCF-7, BT-20 and normal human mammary epithelial cells*

When human dermal fibroblasts were co-cultured with oestrogen-dependent adenocarcinoma MCF-7 cells, oestrogen-independent adenocarcinoma BT-20 cells or normal human mammary epithelial cells, MCF-7 cells enhanced the production of both pro-MMP-1 and -3, whereas BT-20 and normal mammary epithelial cells did not modulate the production of these pro-MMPs significantly (Figure 1a and 1b).

The production of pro-MMP-2 was augmented by both types of adenocarcinoma cells, but not by normal epithelial cells (Figure 1c). The increase in pro-MMP-2 production was further confirmed by gelatin zymography, but pro-MMP-9 was not detected (data not shown). In contrast, TIMP-1 production by dermal fibroblasts was augmented by co-culturing with MCF-7, BT-20 and normal epithelial cells (Figure 1d). These three cell lines did not produce any detectable amounts of pro-MMPs and TIMP-1 (Figures 1a–d, lines 5–7) even when they were treated with human recombinant interleukin 1α or 12-*O*-tetradecanoyl phorbol 13-acetate (data not shown), suggesting that pro-MMPs and TIMP-1 were most probably produced by human dermal fibroblasts. To investigate this further, MCF-7 cells that exerted the greatest effect on the production of MMPs were used for subsequent experiments.

We first examined the effect of the number of MCF-7 cells on the production of pro-MMPs and TIMP-1. When confluent normal fibroblasts were cultured with various cell numbers of MCF-7, the production of pro-MMPs and TIMP-1 coordinately increased in an MCF-7 cell-number dependent manner; the maximum relative increases in pro-MMP-1, -2 and -3 and TIMP-1 were about 3.7-, 4.3-, 2.4- and 1.3-fold respectively (Figure 2). Zymographic analysis indicated that all MMP-2 in the culture media was in a latent form (Figure 2c).

### *Immunolocalisation of MMPs in dermal fibroblasts co-cultured with MCF-7 cells*

To further identify the pro-MMP-producing cells in the above co-culture system, immunohistochemical staining for MMP-1, -2 and -3 was performed. As shown in Figure 3, laser scan microscopy reveals two types of differential interface contact images; one was uniformly small and round or polygonal cells (MCF-7 cells) frequently forming clusters, and the other type was relatively large, flattened and spindle-shaped cells (dermal fibroblasts) (Figure 3b, d and f). The immunofluorescence corresponding to MMP-1, -2 and -3 was localised primarily in the latter fibroblast cells (Figure 3a, c and e). These results further support the hypothesis that the increased production of pro-MMP-1, -2 and -3 by the co-culture resulted predominantly from the activated dermal fibroblasts.

**Figure 4** Effect of the cell membrane of MCF-7 cells on the production of pro-MMPs and TIMP-1 from human dermal fibroblasts. A cell membrane fraction prepared from MCF-7 cells was sonicated in 0.2% (w/v) LAH–MEM to make a homogeneous suspension. Confluent dermal fibroblasts ($12 \times 10^4$ cells) at the 19th passage in 12 multiwell plates were treated with the cell membrane suspension corresponding to $6 \times 10^4$ cells of MCF-7 for 48 h. Microscopic examination of the treated cells indicated uniform spreading of cell membrane debris on fibroblasts. The harvested culture media were subjected to Western blotting and/or gelatin zymography. The relative amounts of each protein produced were quantified as described in Figure 1. (**a**) Pro-MMP-1; (**b**) pro-MMP-3; (**C**) A = zymography and B = Western blotting for pro-MMP-2; (**d**) TIMP-1. Lane 1, untreated human dermal fibroblasts; lane 2, human dermal fibroblasts treated with the MCF-7 cell membrane; lane 3, MCF-7 cell membrane alone.

**Figure 5** Effect of MCF-7 cell conditioned medium on the production of pro-MMPs and TIMP-1 from human dermal fibroblasts. Confluent human dermal fibroblasts ($12 \times 10^4$ cells) at the 18th passage in 12 multiwell plates were treated with concentrated MCF-7 cell conditioned medium obtained from 48 h culture of $6 \times 10^4$ cells in 0.2% (w/v) LAH–MEM for 48 h, and then the harvested culture media were subjected to Western blotting and/or gelatin zymography. The relative amounts of protein produced in each case were quantified and shown as described in Figure 1. (**a**) Pro-MMP-1; (**b**) pro-MMP-3; (**c**) A = pro-MMP-2 zymography and B = Western blotting; (**d**) TIMP-1. Lane 1, untreated human dermal fibroblasts; lane 2, human dermal fibroblasts treated with MCF-7 cell conditioned medium; lane 3, MCF-7 cell conditioned medium alone.

**Figure 6** Effect of MCF-7 cell conditioned medium on the gelatinolytic activity produced by human dermal fibroblasts. Confluent human dermal fibroblasts at the 12th passage were treated with MCF-7 cell conditioned medium as described in Figure 5. The activation of pro-MMP-2 with APMA and assay for gelatinolytic activity in the culture media were done as described in the text. Results are shown as the means ± s.d. of three wells. **Significantly different from untreated cells ($P<0.01$).

*Effect of the MCF-7 cell conditioned medium and the MCF-7 cell membrane on the production of pro-MMPs and TIMP-1 in human dermal fibroblasts*

To investigate whether the pro-MMPs and TIMP-1 production-stimulatory activity of MCF-7 cells is due to a soluble factor secreted by MCF-7 cells or to cell–cell contact, we examined the stimulatory activity in conditioned medium and in cell membranes prepared from MCF-7 cells. When human dermal fibroblasts were treated with cell membranes, the production of pro-MMP-1 and -3 and TIMP-1, but not that of Pro-MMP-2, was augmented (Figure 4), whereas the conditioned medium coordinately augmented the production of all four proteins in the fibroblasts (Figure 5). No significant augmentation of pro-MMP-2 production was observed even when the amount of membrane added to fibroblasts was increased, under which conditions the production of the two other pro-MMPs was greatly enhanced. It is therefore likely that the stimulatory factor(s) secreted in the culture medium is different from that associated with MCF-7 cell membranes.

The apparent MMP activities are controlled by the balance between active MMPs and TIMPs. We, therefore, examined whether the above changes in pro-MMPs and TIMP-1 production caused by the MCF-7 cell-conditioned medium resulted in an increase in apparent MMP activities. As shown in Figure 6, when human dermal fibroblasts were treated with various concentrations of the MCF-7 conditioned medium, the apparent gelatinolytic activity in the culture medium detected after APMA activation increased significantly, indicating that the overall increase in production of pro-MMPs was greater than that of TIMPs.

### Discussion

In this report, we have demonstrated that two adenocarcinoma cell lines which do not produce any significant amount of pro-MMPs augment the production of pro-MMPs by normal stromal cells. When normal dermal fibroblasts were co-cultured with oestrogen-dependent adenocarcinoma MCF-7 cells, the production of pro-MMP-1, -2 and -3 and TIMP-1 in normal fibroblasts was greatly enhanced, whereas oestrogen-independent adenocarcinoma BT-20 cells increased only pro-MMP-2 and TIMP-1. The difference in effect between MCF-7 and BT-20 on the production of pro-MMPs may be attributed to the cell types rather than to the status of oestrogen receptor, since MCF-7 cells cultured in 10% (v/v) oestrogen-depleted FBS–MEM similarly augumented the production of both pro-MMPs and TIMP-1 in fibroblasts (data not shown). It is, furthermore, noteworthy that normal mammary epithelial cells increased only the production of TIMP-1. These results indicate the specific stimulatory effects of breast adenocarcinoma cells on the production of pro-MMPs and TIMP-1 in the normal dermal fibroblasts.

To investigate further the effect of cancer cell–normal cell interaction, we examined the distribution of pro-MMPs and TIMP-1 production stimulatory activity in the cell membrane and the conditioned medium of MCF-7 cells. Our observations indicate that this cell line produces at least two species or sets of stimulatory factors since the conditioned medium augmented the production of pro-MMP-1, -2 and -3 and TIMP-1, whereas the cell membrane enhanced the production of pro-MMP-1, pro-MMP-3 and TIMP-1 but not that of pro-MMP-2. The membrane-bound tumour cell-derived collagenase stimulatory factor (TCSF) was isolated from human lung carcinoma cells by Ellis *et al.* (1989). This factor enhances the expression of pro-MMP-1 and -3 mRNAs in the human colon fibroblast cell line CCD-18 and other human fibroblasts, and it also accelerates the conversion of pro-MMP-2 to an active form (Kataoka *et al.*, 1993). The factor(s) associated with the MCF-7 cell membranes, however, did not influence the activation of pro-MMP-2. Thus, it is likely that the factor(s) in MCF-7 cells is not TCSF.

By contrast, the stimulatory activities found in the conditioned medium of MCF-7 cells appear to be distinct from those on the cell surface. It has been reported that MCF-7 cells produce several growth factors, including transforming growth factor α (TGF-α) (Bates *et al.*, 1988; Kosano *et al.*, 1992), TGF-β (Knabbe *et al.*, 1987) and insulin-like growth factor I (Huff *et al.*, 1986). TGF-α and TGF-β are known to modulate the production of MMPs in several cell types. Indeed, we have observed that a high concentration (> 10 ng $ml^{-1}$) of TGF-α augments the production of pro-MMP-1 and -3 and TIMP-1 without affecting the production of pro-MMP-2 in a dose-dependent manner (A Ito, S Nakajima and Y Mori, unpublished observation). On the other hand, TGF-β at concentrations greater than 100 pg $ml^{-1}$ increases the production of pro-MMP-2 and TIMP-1, but suppresses the production of pro-MMP-1 and -3 in human dermal fibroblasts (A Ito, S Nakajima and Y Mori, unpublished observation). In addition, studies by Kosano *et al.* (1992) and Knabbe *et al.* (1987) suggest that the effective concentrations of these cytokines are higher than the concentrations usually found in the culture media of MCF-7 cells. It is therefore unlikely that TGF-α and/or TGF-β are the primary factors from MCF-7 cells that augmented the production of pro-MMPs and TIMP-1 in human dermal fibroblasts.

The invasive and metastatic properties of MCF-7 cells have been reported, but the relation between these properties and their ability to produce matrix-degrading enzymes has not been clarified (Shafie and Liotta, 1980; Gelmann *et al.*, 1992). Recently, Shi *et al.* (1993) reported that MCF-7 cells produce a novel 80 kDa gelatinolytic metalloprotease which plays a significant role in cancer cell invasion and metastasis when MCF-7 cells are inoculated into nude mice. However, we did not observe any gelatinolytic activities in our zymographic analysis of the MCF-7 conditioned medium. This discrepancy may be due to the different assay conditions, i.e. we examined the gelatinolytic activity using the unconcentrated culture medium of MCF-7 cells, whereas Shi *et al.* (1993) used a 20-fold concentrated medium.

In conclusion, the ability of MCF-7 cells to augment the production of pro-MMPs in surrounding normal fibroblasts is probably one of the important properties for MCF-7 cell invasion and metastasis. Our present data may partly explain why the MMP gene is often predominantly expressed in the stromal cells that surround invasive neoplastic cells (Basset *et al.*, 1990; Pyke *et al.*, 1992). Purification and characterisation of the factor(s) in the conditioned culture medium of MCF-7 cells are now in progress.

**Acknowledgements**
We are grateful to Dr Hiroshi Kosano (Teikyo University, Kanagawa, Japan) for generously providing us with MCF-7 and BT-20

cell lines and for his helpful discussion. We also thank Dr Toshiaki Takezawa (Grace Japan Co., Kanagawa, Japan) for kindly providing us with human normal dermal fibroblasts. This work was supported in part by a grant from the Cosmetology Research Foundation.

### References

ALBINI A, MELCHIORI A, SATI L, LIOTTA L, BROWN PD AND STETLER-STEVENSON WG. (1991). Tumor cell invasion inhibited by TIMP-2. *J. Natl Cancer Inst.*, **83**, 775–779.

BAICI A, GYGER-MARAZZI M AND STRAULI P. (1984). Extracellular cysteine proteinase and collagenase activities. *Invasion Metast.*, **4**, 13–27.

BASSET P, BELLOCQ JP, WOLF C, STOLL I, HUTIN P, LIMACHER JM, PODHAJCER OL, CHENARD MP, RIO MC AND CHAMBON P. (1990). A novel metalloproteinase gene specifically expressed in stromal cells of breast carcinoma. *Nature*, **348**, 699–704.

BATES SE, DAVIDSON NE, VALVERIUS EM, FRETER CE, DICKSON RB, TAM JP, KUDLOW JE, LIPPMAN ME AND SALOMON DS. (1988). Expression of transforming growth factor-α and its messenger ribonucleic acid in human breast cancer: its regulation by estrogen and its possible functional significance. *Mol. Endocrinol.*, **2**, 543–555.

BAYLIN M, GOME3Z E, SINHA CC AND THORGEIRSSON UP. (1988). Ras oncogene mediated induction of a 92-kDa metalloproteinase: strong correlation with the malignant phenotype. *Biochem. Biophys. Res. Commun.*, **154**, 832–838.

BERNHARD EJ, MUSCHEL RJ AND HUGHES EN. (1990). *Mr* 92,000 gelatinase release correlates with the metastatic phenotype in transformed rat embryo cells. *Cancer Res.*, **50**, 3872–3877.

BISWAS C. (1982). Tumor cell stimulation of collagenase production by fibroblasts. *Biochem. Biophys. Res. Commun.*, **109**, 1026–1034.

BISWAS C. (1984). Collagenase stimulation in cocultures of human fibroblasts and human tumor cells. *Cancer Lett.*, **24**, 201–207.

BISWAS C AND NUGENT MA. (1987). Membrane association of collagenase stimulatory factor(s) from B-16 melanoma cells. *J. Cell. Biochem.*, **35**, 247–258.

BISWAS C, BLOCH KJ AND GROSS J. (1982). Collagenolytic activity of rabbit V2 carcinoma implanted on the nude mouse. *J. Natl Cancer Inst.*, **69**, 1329–1336.

DABBOUS MK, EL-TORKY M, HANEY L, BRINKLEY B AND SOBHY N. (1983). Collagenase activity in rabbit carcinoma: cell source and interaction. *Int. J. Cancer*, **31**, 357–364.

DECLERCK YA, YEAN TS, CHAN D, SHIMADA H AND LANGLEY KE. (1991). Inhibition of tumour invasion of smooth muscle cell layers by recombinant human metalloproteinase inhibitor. *Cancer Res.*, **51**, 2151–2157.

DECLERCK YA, PEREZ N, SHIMADA H, BOONE TC, LANGLEY KE AND TAYLOR SM. (1992). Inhibition of invasion and metastasis in cells transfected with an inhibitor of metalloproteinases. *Cancer Res.*, **52**, 701–708.

ELLIS SM, NABESHIMA K AND BISWAS C. (1989). Monoclonal antibody preparation and purification of a tumor cell collagenase-stimulatory factor. *Cancer Res.*, **49**, 3385–3391.

GABRISA S, POZZATTI R, MUSCHEL RJ, SAFFIOTTI U, BALLIAN M, GOLDFARB RH, KHOURY G AND LIOTTA LA. (1987). Secretion of type IV collagenolytic protease and metastatic phenotype: induction by transfection with C-Ha-ras but not C-Ha-ras plus Ad2-Ela. *Cancer Res.*, **47**, 1523–1528.

GELMANN EP, THOMPSON EW AND SOMMERS CL. (1992). Invasive and metastatic properties of MCF-7 cells and $ras^H$-transfected MCF-7 cell lines. *Int. J. Cancer*, **50**, 665–669.

GOLSLEN JB, EISEN AZ AND BAUER EA. (1985). Stimulation of skin fibroblast collagenase production by a cytokine derived from basal cell carcinoma. *J. Invest. Dermatol.*, **85**, 161–164.

HUFF KK, KAUFMAN D, GABBAY KH, SPENCER EM, LIPPMAN ME AND DICKSON RB. (1986). Secretion of an insulin-like growth factor-I-related protein by human breast cancer cells. *Cancer Res.*, **46**, 4613–4619.

IMADA K, ITO A, ITOH Y, NAGASE H AND MORI Y. (1994). Progesterone increases the production of tissue inhibitor of metalloproteinases-2 in rabbit uterine cervical fibroblasts. *FEBS Lett.*, **341**, 109–112.

ISHIBASHI M, ITO A, SAKYO K AND MORI Y. (1987). Procollagenase activator produced by rabbit uterine cervical fibroblasts. *Biochem. J.*, **241**, 527–534.

ITO A AND NAGASE H. (1988). Evidence that human rheumatoid synovial matrix metalloproteinase 3 is an endogenous activator of procollagenase. *Arch. Biochem. Biophys.*, **267**, 211–216.

KATAOKA H, DECASTRO R, ZUCKER S AND BISWAS C. (1993). Tumor cell-derived collagenase-stimulatory factor increases expression of interstitial collagenase, stromelysin and 72-kDa gelatinase. *Cancer Res.*, **53**, 3154–3158.

KNABBE C, LIPPMANN ME, WAKEFIELD LM, FLANDERS KC, KASID A, DERYNCK R AND DICKSON RB. (1987). Evidence that transforming growth factor-β is a hormonally regulated negative growth factor in human breast cancer cells. *Cell*, **48**, 417–428.

KOSANO H AND TAKATANI O. (1988). Reduction of epidermal growth factor binding in human breast cancer cell lines by an alkyl-lysophospholipid. *Cancer Res.*, **48**, 6033–6036.

KOSANO H AND TAKATANI O. (1989). Inhibition by an alkyl-lysophosholipid of the uptake of epidermal growth factor (EGF) in human breast cancer cell lines in relation to EGF internalization. *Cancer Res.*, **49**, 2868–2870.

KOSANO H, KUBOTA T, OHSAWA N, YAMAMORI S, ABE O, INAGAKI H AND NAGATA N. (1992). Growth-inhibitory action of an estrogen–chlorambucil conjugate (KM2210) in human breast cancer cell line MCF-7: its regulation of reduction of estrogen receptor and transforming growth factor-α secretion. *Cancer Res.*, **52**, 1187–1191.

LAEMMLI UK. (1970). Cleavage of structural proteins during the assembly of the head of bacteriophage T4. *Nature*, **227**, 680–685.

LIOTTA LA AND STETLER-STEVENSON WG. (1989). Metalloproteinases and malignant conversion: does correlation imply causality? *J. Natl Cancer Inst.*, **81**, 556–557.

LIOTTA LA AND STETLER-STEVENSON WG. (1991). Tumor invasion and metastasis: an imbalance of positive and negative regulation. *Cancer Res.*, **51** (Suppl.), 5054s–5059s.

MATRISIAN LM AND BOWDEN Y. (1990). Stromelysin/transin and tumor progression. *Semin. Cancer Biol.*, **1**, 107–115.

MONTEAGUDO C, MERINO MJ, SAN-JUAN J, LIOTTA LA AND STETLER-STEVENSON WG. (1990). Immunohistochemical distribution of type IV collagenase in normal, benign and malignant breast tissue. *Am. J. Pathol.*, **136**, 585–592.

MURPHY G, SEGAIN J-P, O'SHERA M, COCHETT M, IOANNOU C, LEFEBVRE O, CHAMBON P AND BASSET P. (1993). The 28-kDa N-terminal domain of mouse stromelysin 3 has the general properties of a weak metalloproteinase. *J. Biol. Chem.*, **268**, 15435–15441.

NAKAJIMA M, WELCH DR, BELLONI PN AND NICOLSON GL. (1987). Degradation of basement membrane type IV collagen and lung subendothelial matrix by rat mammary adenocarcinoma cell clones of differing metastatic potentials. *Cancer Res.*, **47**, 4869–4876.

OJIMA Y, ITO A, NAGASE H AND MORI Y. (1989). Calmodulin regulates the interleukin 1-induced procollagenase production in human uterine cervical fibroblasts. *Biochim. Biophys. Acta*, **1011**, 61–66.

OKADA Y, TSUCHIYA H, SHIMIZU H, TOMITA K, NAKANISHI I, SATO H, SEIKI M, YAMASHITA K AND HAYAKAWA T. (1990). Induction and stimulation of 92-kDa gelatinase/type IV collagenase production in osteosarcoma and fibrosarcoma cell lines by tumor necrosis factor α. *Biochem. Biophys. Res. Commun.*, **171**, 610–617.

PRESCOTT J, TROCCOLI N AND BISWAS C. (1989). Coordinate increase in collagenase mRNA and enzyme levels in human fibroblasts treated with the tumor cell factor, TCSF. *Biochem. Int.*, **19**, 257–266.

PYKE C, RALFKIAER E, HUHTALA P, HURSKAINEN T, DANO K AND TRYGGVASON K. (1992). Localization of messenger RNA for Mr 72,000 and 92,000 type IV collagenase in human skin cancers by *in situ* hybridization. *Cancer Res.*, **52**, 1336–1341.

SASAGURI Y, KOMIYA S, SUGAMA K, SUZUKI K, INOUE A, MORIMATSU M AND NAGASE H. (1992). Production of matrix metalloproteinase 2 and 3 (stromelysin) by stromal cells of giant cell tumor of bone. *Am. J. Pathol.*, **141**, 611–621.

SHAFIE SM AND LIOTTA LA. (1980). Formation of metastasis by human breast carcinoma cells (MCF-7) in nude mice. *Cancer Lett.*, **11**, 81–87.

SHI YE, TORRI J, YIEH L, WELLSTEIN A, LIPPMAN ME AND DICKSON RE. (1993). Identification and characterization of a novel matrix-degradation proteases from hormone-dependent human breast cancer cells. *Cancer Res.*, **53**, 1409–1415.

SHULTZ RM, SILBERMAN S, PERSKY B, BAJKOWSKI AS AND CARMICHAEL DF. (1988). Inhibition by human recombinant tissue inhibitor of metalloproteinase of human amnion invasion and lung colonization by murine B16-F10 melanoma cells. *Cancer Res.*, **48**, 5539–5545.

TAKAHASHI S, SATO T, ITO A, OJIMA Y, HOSONO T, NAGASE H AND MORI Y. (1993). Involvement of protein kinase C in the interleukin 1α-induced gene expression of matrix metalloproteinases and tissue inhibitor-1 of metalloproteinases (TIMP-1) in human uterine cervical fibroblasts. *Biochim. Biophys. Acta*, **1220**, 57–65.

URA H, BONFIL D, REICH R, REDDEL R, PFEIFER A AND HARRIS CC. (1989). Expression of type IV collagenase and procollagen genes and its correlation with tumorigenic invasive, and metastatic abilities of oncogene-transformed human bronchial epithelial cells. *Cancer Res.*, **49**, 4615–4621.

WILHELM SM, COLLER IE, MARMER BL, EISEN AZ, GRANT GA AND GOLDBERG GI. (1989). SV40-transformed human lung fibroblasts secrete a 92-kDa type IV collagenase which is identical to that secreted by normal macrophages. *J. Biol. Chem.*, **264**, 17213–17222.

YAMAGATA S, TANAKA R, YOSHIDA I AND SHIMIZU S. (1989). Gelatinase of murine metastatic tumor cells. *Biochem. Biophys. Res. Commun.*, **158**, 228–234.